# Special Issue “Molecular Insights into Zoology”

**DOI:** 10.3390/ijms27209236

**Published:** 2026-10-17

**Authors:** Valeria Maselli

**Affiliations:** Department of Biology, University of Naples Federico II, 80126 Naples, Italy; valeria.maselli@unina.it

Zoology has entered an era in which molecular approaches are no longer ancillary tools, but central components to grasp animal diversity, evolution, physiology, adaptation, and conservation. Modern zoological research increasingly depends on integrative molecular frameworks that connect genotype, phenotype, environment, and evolutionary history, from mitochondrial genomics to transcriptomics and from molecular diagnostics to conservation genomics. The Special Issue “Molecular Insights into Zoology” was conceived within this perspective to bring together studies applying molecular methods throughout diverse animal systems and biological questions, from vertebrates to invertebrates, terrestrial to aquatic organisms, and wild to farmed species, spanning both basic and applied research. Despite this diversity, molecular tools allow researchers to move beyond descriptive observation toward mechanistic, diagnostic, and predictive interpretations of animal biology.

This transformation is occurring within a broader expansion of genomic resources across the tree of life. In particular, large-scale initiatives are accelerating the generation of reference genomes for non-model organisms [1], creating unprecedented opportunities for comparative zoology, evolutionary biology, and biodiversity conservation [2,3].

A first major theme of this Special Issue is the use of mitochondrial genomes and mitochondrial markers to reconstruct evolutionary relationships, assess genetic diversity, and infer adaptation. Several contributions demonstrate how complete mitogenomes can clarify lineage diversification, phylogenetic placement, codon usage evolution, and selective pressures across very different zoological groups, including mammals, insects, and marine fishes.

Mitochondrial DNA remains particularly informative in zoological research because its biological and genomic features make it suitable for studies ranging from species identification to population genetics and deep evolutionary reconstruction. In most animals, the predominantly maternal inheritance of mtDNA, its generally limited recombination and relatively rapid sequence evolution, together with its high cellular copy number, make it particularly informative for reconstructing matrilineal histories and detecting genetic differentiation, including from limited or degraded biological material [4]. Moreover, the mitochondrial genome combines protein-coding genes, ribosomal and transfer RNA genes, and non-coding regions that evolve under different functional constraints and therefore provide complementary information across evolutionary timescales.

The progressive reduction in sequencing costs, together with advances in high-throughput sequencing and bioinformatic approaches, has substantially expanded the scope of mitochondrial studies [5]. Analyses that were historically based on individual loci or short mitochondrial fragments can now progressively depend on complete mitogenomes, including population-level datasets comprising multiple complete mitochondrial sequences [6]. This transition provides greater phylogenetic resolution and enables genetic diversity, demographic history, population structure, and signatures of selection to be investigated across the entire mitochondrial genome rather than inferred from a limited number of markers [6,7]. Importantly, complete mitogenomes also provide information beyond nucleotide variation itself. Gene order and orientation, intergenic regions, gene overlaps, duplications and rearrangements, codon usage, nucleotide composition, and control-region structural features can all retain evolutionary signals and reveal lineage-specific patterns that would remain undetected in single-marker analyses [8,9]. Comparative mitogenomics therefore transforms mitochondrial DNA from a convenient molecular marker into an integrated genomic system for examining sequence evolution, genome architecture, population history, and adaptation throughout a common evolutionary framework [10].

In particular, Wang et al. [11] analyzed complete mitogenomes of Xinjiang hares and explored selective pressure across mitochondrial genes, supplying insights into the evolutionary dynamics of *Lepus* species inhabiting contrasting environments. Their work suggests that mitochondrial variation, codon usage, and selection signals in genes such as *ND4* and *ND5* may be associated with adaptation to extreme ecological conditions, including arid, hot, cold, and high-altitude environments.

Beyond individual case studies, recent comparative work confirms that researchers increasingly use complete mitochondrial genomes for species identification, phylogenetic reconstruction, and analysis of genome architecture across Metazoa. It also reveals non-canonical mitochondrial organizations that broaden the evolutionary questions accessible through mitogenomic data [12,13].

Mitochondrial genomics is also central in the work of Kundu et al. [14], who investigated the complete mitochondrial genome of the Atlantic emperor, *Lethrinus atlanticus*, an Eastern Atlantic species. Their study described the mitogenomic architecture of this reef-associated fish, including the organization of 37 mitochondrial genes, codon usage patterns, conserved control-region domains, and selective pressure across protein-coding genes. The evidence of strong negative selection across mitochondrial PCGs and the phylogenetic reconstruction of Spariformes provide important knowledge of the evolutionary stability and lineage diversification of emperor fishes. Notably, the estimated divergence of *L. atlanticus* from its Indo-West Pacific congeners during the late Oligocene highlights how oceanographic history, ecological conditions, and geographic separation may have affected Lethrinidae diversification across these distinct marine biomes.

Beyond these specific examples, expanding taxonomic and geographic sampling remains essential for interpreting mitochondrial diversity in animals. Increased access to complete mitogenomes from native populations, museum collections, and previously underrepresented lineages is improving comparative resolution and also highlights the need for careful sampling design and for integrating mitochondrial evidence with independent genomic and biological information [5,13].

Despite their considerable value, the inherent limitations of mitochondrial data should also be carefully considered when interpreting evolutionary and population patterns. Mitochondrial genomes represent only the maternally inherited component of animal genetic history, and mitochondrial and nuclear patterns may not always be concordant. In the current genomic era, cytonuclear discordance is increasingly recognized not simply as a methodological complication, but as a biologically informative pattern that may reflect distinct evolutionary histories of mitochondrial and nuclear genomic compartments [15]. Reliance on mitochondrial data alone may also provide an incomplete picture of interspecific hybridization and introgression, because uniparental inheritance traces only the maternal lineage and may fail to reveal the biparental genomic ancestry of hybrid individuals [16]. Conversely, mitochondrial introgression or mitochondrial capture may obscure species boundaries and generate misleading interpretations when mitochondrial markers are considered in isolation [17,18]. Integrating mitochondrial data with nuclear genomic evidence is therefore essential for distinguishing these evolutionary processes and obtaining a more comprehensive reconstruction of species and population histories, representing an important direction for future research [16,17,18]. Recent phylogenomic studies clearly illustrate the relevance of this approach; for instance, Lawson et al. [19] showed that combining mitochondrial and genome-wide nuclear data in East African reed frogs revealed mitonuclear discordance and previously unrecognized evolutionary lineages, with important implications for species delimitation and biodiversity conservation.

The value of integrating mitochondrial and nuclear genomic information becomes particularly evident in conservation genetics and genomics, where complementary maternal and biparental data can provide a more comprehensive understanding of population and reproductive dynamics. Gaspar et al. [20] combined mitochondrial tandem repeat-region profiling with genome-wide SNP data obtained through 3RADseq to investigate the Mediterranean population of the critically endangered Nile soft-shelled turtle, *Trionyx triunguis*. Their study revealed complex reproductive dynamics, including multiple paternity, sperm storage, and repeated nesting within a single season. By combining these complementary markers, the authors provided a genomic framework for monitoring population structure and reproductive dynamics, with direct relevance to conservation management. In a broader context, population genomics is increasingly providing conservation biology with tools to identify management units, estimate connectivity and effective population size, detect inbreeding and hybridization, and assess adaptive potential [21,22,23,24]. However, a key gap remains between generating genomic information and implementing it routinely in conservation decision-making. Bridging this research-to-practice divide will require closer collaboration among scientists, wildlife managers, and policy makers [21,22,23,24].

Molecular diagnostics provide another important application in conservation. Norcia et al. [25] developed and validated a High-Resolution Melting analysis protocol for the rapid and cost-effective identification of four representative Mediterranean cetacean species: *Tursiops truncatus*, *Stenella coeruleoalba*, *Physeter macrocephalus*, and *Balaenoptera physalus*. The method was successfully applied to both tissue and fecal samples and showed high sensitivity, reproducibility, and discrimination power. This contribution illustrates how molecular diagnostics can support non-invasive monitoring, wildlife forensics, and the enforcement of conservation regulations, particularly when morphological identification is difficult or impossible. Importantly, this application also highlights how the extensive knowledge of mitochondrial markers, together with the growing availability of mitochondrial sequences in public databases, can be translated into effective tools for applied zoological research.

Molecular surveillance goes beyond simply identifying species. Using DNA to detect hosts, pathogens, and parasites, along with their interactions, can improve sensitivity over traditional methods based on morphology or observation [26]. This approach supports targeted management, especially when organisms are cryptic, rare, or hard to sample. These applications increasingly overlap with environmental DNA and other non-invasive molecular monitoring strategies [27,28,29,30,31,32].

These developments also align with the rapid expansion of environmental DNA (eDNA)-based approaches, which are transforming biodiversity surveillance by enabling sensitive, non-invasive detection of species and communities directly from environmental samples [30]. Despite their potential, broader implementation will require improved methodological standardization, more complete reference databases, and careful validation across taxonomic groups and environmental contexts [33,34].

Overall, the growing power of molecular approaches should not lead to molecular identification being treated as a replacement for traditional morphological taxonomy. Whenever possible, molecular evidence should be integrated with morphological identification and complemented by ecological, biogeographic, behavioral, and other relevant biological information within an integrative taxonomic framework [35,36].

Another central axis of the Special Issue is functional genomics and transcriptomics as tools for understanding animal responses to environmental pressures. Han et al. [37] investigated the effects of exposure to the harmful algal bloom-forming dinoflagellate *Alexandrium pacificum* on the shrimp *Exopalaemon carinicauda*. Through histological analysis, antioxidant enzyme assays, and transcriptomic profiling, they demonstrated that algal exposure induces hepatopancreatic damage, oxidative stress, and major transcriptional changes involving pathways related to endoplasmic reticulum protein processing, mitophagy, glycolysis/gluconeogenesis, sphingolipid metabolism, and glycerophospholipid metabolism. This study provides mechanistic insight into the ecotoxicological consequences of harmful algal blooms in aquatic organisms and aquaculture systems.

Evidence from other aquatic species shows that RNA-seq can reveal recurrent molecular responses to environmental stress, including oxidative stress, immune and inflammatory pathways, xenobiotic metabolism, and cellular homeostasis, thereby helping to connect exposure with mechanism [38,39].

Yévenes et al. [40] addressed local adaptation in the Chilean blue mussel *Mytilus chilensis* through a reciprocal transplant experiment combined with transcriptomic and genomic analyses. Their results illustrate that natural seedbeds from ecologically distinct environments retain signatures of local adaptation, notwithstanding potential homogenizing effects associated with larval dispersal and aquaculture-related translocations. Differentially expressed genes and outlier SNP-associated regions were linked to immune function, osmoregulation, oxidative stress, oxygen management, and cellular homeostasis, highlighting the importance of preserving locally adapted populations and their genomic diversity for sustainable aquaculture and conservation management.

Molecular tools are becoming more important for animal management, breeding, and health. In captive, farmed, and managed populations, genomic and multi-omic techniques help with sex determination, reconstructing parentage and pedigrees, identifying functional biomarkers, evaluating disease resistance, and analyzing traits related to reproduction and production. Their effectiveness is highest when molecular data is combined with phenotype, physiology, and management practices [41,42].

At the functional level, integrating genomics, transcriptomics, proteomics, metabolomics, and epigenomics is progressively shifting animal research from single molecular markers toward systems-level interpretations of complex phenotypes. Such approaches can connect regulatory variation with cellular and organismal responses, although data integration, computational complexity, sample size, and cross-study standardization remain important limitations [42,43].

Another important message emerging from this collection is the increasing convergence between basic and applied zoology. Molecular data are not only useful for reconstructing evolutionary histories; they also inform conservation planning, aquaculture sustainability, disease control, wildlife forensics, animal breeding, and environmental risk assessment.

Despite these advances, significant challenges remain. Genomic resources remain unevenly distributed across animal taxa and geographic regions, and many non-model and threatened taxa remain poorly characterized. Molecular signals also require stronger integration with ecological, phenotypic, physiological, demographic, and environmental data. Moreover, the growing availability of genomic information has not yet been fully matched by its incorporation into conservation and management practice, underscoring the need to strengthen communication among molecular researchers, ecologists, wildlife managers, conservation practitioners, and decision-makers [22,23,24,44].

Taken together, the articles published in this Special Issue demonstrate that molecular zoology is both conceptually broad and methodologically powerful (Figure 1).

Future molecular zoology will increasingly rely on integrative approaches that connect multiple levels of biological organization. Multi-omic frameworks that integrate genomics, transcriptomics, epigenomics, proteomics, and metabolomics with environmental DNA, long-term ecological monitoring, and experimental validation will increasingly enable mechanistic and predictive analyses of animal responses [42,43]. At the same time, broader taxonomic and geographic sampling, improved reference genomes and databases, standardized analytical frameworks, and open, interoperable molecular resources will be essential to ensure these advances extend beyond a limited number of well-characterized species [2,27,33,44].

Moreover, artificial intelligence and machine learning approaches are expected to further expand the analytical and predictive potential of zoological research. AI-assisted frameworks may facilitate the integration of genomic, morphological, ecological, and phenotypic evidence, improve species delimitation and biodiversity assessment, and help identify complex patterns that may remain difficult to detect using individual data sources or conventional analytical approaches [35,45]. AI should complement integrative taxonomy by supporting more robust, scalable, and reproducible approaches to the characterization and interpretation of animal diversity [46,47].

## Figures and Tables

**Figure 1:**
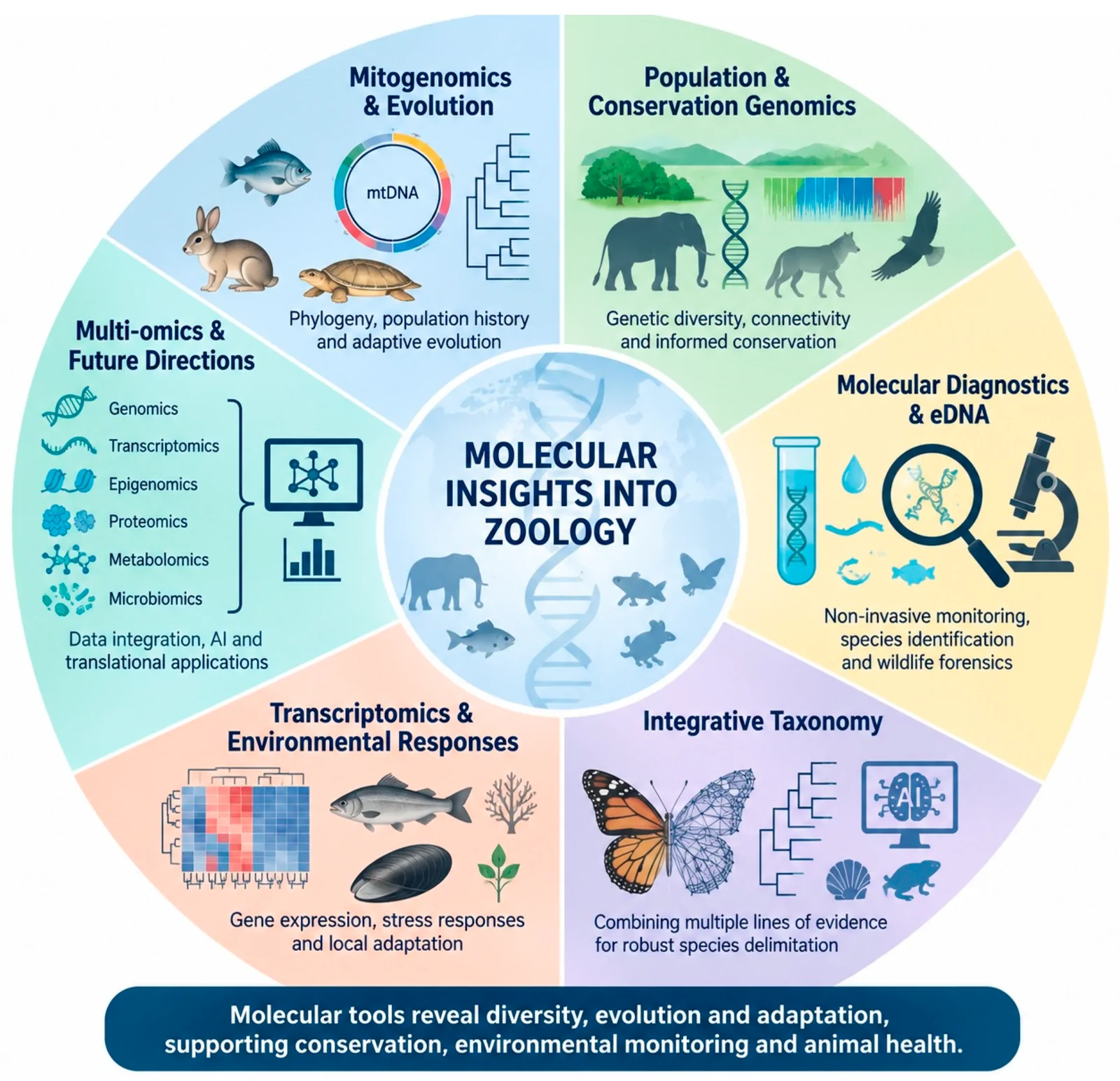
Infographic depicting the main molecular approaches and emerging applications in zoological research (note: figure generated using ChatGPT (OpenAI, GPT-5.6) and adapted by the author).

## References

[1] Kosch T.A., Torres-Sánchez M., Liedtke H.C., Summers K., Yun M.H., Crawford A.J., Maddock S.T., Ahammed M.S., Araújo V.L.N., Bertola L.V. (2024). The Amphibian Genomics Consortium: Advancing genomic and genetic resources for amphibian research and conservation. BMC Genom..

[2] Blaxter M., Lewin H.A., DiPalma F., Challis R., da Silva M., Durbin R., Formenti G., Franz N., Guigo R., Harrison P.W. (2025). The Earth BioGenome Project Phase II: Illuminating the eukaryotic tree of life. Front. Sci..

[3] Theissinger K., Fernandes C., Formenti G., Bista I., Berg P.R., Bleidorn C., Bombarely A., Crottini A., Gallo G.R., Godoy J.A. (2023). How genomics can help biodiversity conservation. Trends Genet..

[4] Veeraragavan S., Johansen M., Johnston I.G. (2024). Evolution and maintenance of mtDNA gene content across eukaryotes. Biochem. J..

[5] Zhu Y.-W., He M., Wang H.-S., Liang D., Zhang P. (2025). A comparison and evaluation of strategies for bulk sequencing of mitogenomes from museum collections. Methods Ecol. Evol..

[6] Vieira A.R., de Sousa F., Bilro J., Viegas M.B., Svanbäck R., Gordo L.S., Paulo O.S. (2024). Mitochondrial genomes of the European sardine (*Sardina pilchardus*) reveal Pliocene diversification, extensive gene flow and pervasive purifying selection. Sci. Rep..

[7] Jansamut P., Gale G.A., Sukmak M., Chimchome V., Klinsawat W. (2025). Population mitogenome diversity and connectivity among wild populations of Great Hornbill (*Buceros bicornis*) in Thailand: Implications for population recovery planning. Glob. Ecol. Conserv..

[8] Sterling-Montealegre R.A., Prada C.F. (2024). Variability and evolution of gene order rearrangement in mitochondrial genomes of arthropods (except Hexapoda). Gene.

[9] Zhang Y., Heng X., Li F., Liu C., Li Q., Wang A., Yang Y. (2025). Comparative mitogenomic analyses of the infraclass Pteriomorphia (Mollusca: Bivalvia) provides novel insights into gene rearrangement and phylogeny. Comp. Biochem. Physiol. D Genom. Proteom..

[10] Jakovlić I., Ma Y.-W., Ye T., Han R., Liu X., Xiang C.-Y., Zou H., Zhang J., Song R., Li W.-X. (2026). Patterns and determinants of mitogenomic evolution in Bilateria. Nat. Commun..

[11] Wang R., Tursun M., Shan W. (2024). Complete Mitogenomes of Xinjiang Hares and Their Selective Pressure Considerations. Int. J. Mol. Sci..

[12] Xing B., Lin L., Wu Q. (2025). Application of mitochondrial genomes to species identification and evolution. Electron. J. Biotechnol..

[13] Kayal E., Lavrov D.V. (2025). One Ring does not rule them all: Linear mtDNA in Metazoa. Gene.

[14] Kundu S., Kang H.-E., Go Y., Bang G., Jang Y., Htoo H., Aini S., Kim H.-W. (2024). Mitogenomic Architecture of Atlantic Emperor *Lethrinus atlanticus* (Actinopterygii: Spariformes): Insights into the Lineage Diversification in Atlantic Ocean. Int. J. Mol. Sci..

[15] Larson D.A., Itgen M.W., Denton R.D., Hahn M.W. (2026). Reconsidering cytonuclear discordance in the genomic age. Evolution.

[16] de Sá F.P., Akopyan M., Santana E.M., Haddad C.F.B., Zamudio K.R. (2024). Mitonuclear and phenotypic discordance in an Atlantic Forest frog hybrid zone. Ecol. Evol..

[17] Magalhães F.d.M., Oliveira E.F., Garda A.A., Burbrink F.T., Gehara M. (2025). Genomic data support reticulate evolution in whiptail lizards from the Brazilian Caatinga. Mol. Phylogenet. Evol..

[18] Read W.J., Laver R.J., Lau C.C., Moritz C., Zozaya S.M. (2025). Repeated Mitochondrial Capture With Limited Genomic Introgression in a Lizard Group. Mol. Ecol..

[19] Lawson L.P., Bittencourt-Silva G.B., Conradie W., Portik D.M., Loader S.P. (2025). A phylogenomic perspective reveals mitochondrial-nuclear discordance and previously undescribed species nested within a widespread East African Reed frog species (*Hyperolius substriatus* Ahl, 1931). PLoS ONE.

[20] Gaspar A., Arantes L.S., Ohana T., Bodenheimer Y.E., Tikochinski G., Levy O., Mor B.J., Vainberg M., Gat T., Mbedi S. (2025). The Mediterranean Habitat of the Nile Soft-Shelled Turtle (*Trionyx triunguis*): Genomic and Reproductive Insights into an Endangered Population. Int. J. Mol. Sci..

[21] Ghildiyal K., Nayak S.S., Rajawat D., Sharma A., Chhotaray S., Bhushan B., Dutt T., Panigrahi M. (2023). Genomic insights into the conservation of wild and domestic animal diversity: A review. Gene.

[22] Hogg C.J. (2024). Translating genomic advances into biodiversity conservation. Nat. Rev. Genet..

[23] Hohenlohe P.A., Funk W.C., Rajora O.P. (2021). Population genomics for wildlife conservation and management. Mol. Ecol..

[24] Schiebelhut L.M., Guillaume A.S., Kuhn A., Schweizer R.M., Armstrong E.E., Beaumont M.A., Byrne M., Cosart T., Hand B.K., Howard L. (2024). Genomics and conservation: Guidance from training to analyses and applications. Mol. Ecol. Resour..

[25] Norcia M., Illiano A., Mussi B., Di Nocera F., Esposito E., Di Cosmo A., Fulgione D., Maselli V. (2025). When DNA Tells the Tale: High-Resolution Melting as a Forensic Tool for Mediterranean Cetacean Identification. Int. J. Mol. Sci..

[26] Bass D., Christison K.W., Stentiford G.D., Cook L.S.J., Hartikainen H. (2023). Environmental DNA/RNA for pathogen and parasite detection, surveillance, and ecology. Trends Parasitol..

[27] Altermatt F., Couton M., Carraro L., Keck F., Lawson-Handley L., Leese F., Zhang X., Zhang Y., Blackman R.C. (2025). Utilizing aquatic environmental DNA to address global biodiversity targets. Nat. Rev. Biodivers..

[28] Broadhurst H.A., Sales N.G., Raynor R., Howe C., Ochu E., Lambin X., Sutherland C.S., McDevitt A.D. (2025). From Water to Land: A Review on the Applications of Environmental DNA and Invertebrate-Derived DNA for Monitoring Terrestrial and Semi-Aquatic Mammals. Mammal. Rev..

[29] Çevik T., Çevik N. (2025). Environmental DNA (eDNA): A review of ecosystem biodiversity detection and applications. Biodivers. Conserv..

[30] Takahashi M., Saccò M., Kestel J.H., Nester G., Campbell M.A., van der Heyde M., Heydenrych M.J., Juszkiewicz D.J., Nevill P., Dawkins K.L. (2023). Aquatic environmental DNA: A review of the macro-organismal biomonitoring revolution. Sci. Total Environ..

[31] Cowgill C., Gilbert J.D.J., Convery I., Lawson Handley L. (2025). Monitoring terrestrial rewilding with environmental DNA metabarcoding: A systematic review of current trends and recommendations. Front. Conserv. Sci..

[32] Sahu A., Singh M., Amin A., Malik M.M., Qadri S.N., Abubakr A., Teja S.S., Dar S.A., Ahmad I. (2025). A systematic review on environmental DNA (eDNA) Science: An eco-friendly survey method for conservation and restoration of fragile ecosystems. Ecol. Indic..

[33] Takahashi M., Frøslev T.G., Paupério J., Thalinger B., Klymus K., Helbing C.C., Villacorta-Rath C., Silliman K., Thompson L.R., Jungbluth S.P. (2025). A Metadata Checklist and Data Formatting Guidelines to Make eDNA FAIR (Findable, Accessible, Interoperable, and Reusable). Environ. DNA.

[34] Curto M., Veríssimo A., Riccioni G., Santos C.D., Ribeiro F., Jentoft S., Alves M.J., Gante H.F. (2025). Improving Whole Biodiversity Monitoring and Discovery With Environmental DNA Metagenomics. Mol. Ecol. Resour..

[35] Karbstein K., Kösters L., Hodač L., Hofmann M., Hörandl E., Tomasello S., Wagner N.D., Emerson B.C., Albach D.C., Scheu S. (2024). Species delimitation 4.0: Integrative taxonomy meets artificial intelligence. Trends Ecol. Evol..

[36] Kollár J. (2026). Integration, not replacement: Molecular methods and a new equilibrium in taxonomy. Bioscience.

[37] Han W., Cheng W., Fan M., Liu D., Cao Y., Mei X., Wan J., Hu G., Gao H., Ji N. (2025). Effects of *Alexandrium pacificum* Exposure on *Exopalaemon carinicauda*: Hepatopancreas Histology, Antioxidant Enzyme Activity, and Transcriptome Analysis. Int. J. Mol. Sci..

[38] Ahi E.P., Lindeza A.S., Miettinen A., Primmer C.R. (2025). Transcriptional responses to changing environments: Insights from salmonids. Rev. Fish Biol. Fish..

[39] Hechler R.M., Yates M.C., Chain F.J.J., Cristescu M.E. (2025). Environmental transcriptomics under heat stress: Can environmental RNA reveal changes in gene expression of aquatic organisms?. Mol. Ecol..

[40] Yévenes M., Gajardo G., Gallardo-Escárate C. (2025). Decoding Local Adaptation in the Exploited Native Marine Mussel *Mytilus chilensis*: Genomic Evidence from a Reciprocal Transplant Experiment. Int. J. Mol. Sci..

[41] Choudhary R.K., Kumar B.V.S., Sekhar Mukhopadhyay C., Kashyap N., Sharma V., Singh N., Salajegheh Tazerji S., Kalantari R., Hajipour P., Singh Malik Y. (2024). Animal Wellness: The Power of Multiomics and Integrative Strategies: Multiomics in Improving Animal Health. Vet. Med. Int..

[42] Wadood A.A., Bordbar F., Zhang X. (2025). Integrating omics approaches in livestock biotechnology: Innovations in production and reproductive efficiency. Front. Anim. Sci..

[43] Li B., Zhang Y., Du J., Liu C., Zhou G., Li M., Yan Z. (2025). Application of Multi-Omics Techniques in Aquatic Ecotoxicology: A Review. Toxics.

[44] Schmidt T.L., Thia J.A., Hoffmann A.A. (2024). How Can Genomics Help or Hinder Wildlife Conservation?. Annu. Rev. Anim. Biosci..

[45] Oliveira M.B., Bernardino H.S., Vieira A.B., Augusto D.A. (2025). Classification of animal species via deep neural networks and species distribution modeling: A systematic review. Artif. Intell. Rev..

[46] Zhang Q., Ahmed K., Sharda N., Wang H. (2024). A Comprehensive Survey of Animal Identification: Exploring Data Sources, AI Advances, Classification Obstacles and the Role of Taxonomy. Int. J. Intell. Syst..

[47] Kösters L.M., Karbstein K., Hofmann M., Hodač L., Mäder P., Wäldchen J. (2025). Data Fusion for Integrative Species Identification using Deep Learning. Syst. Biol..

